# Technology to accelerate pangenomic scanning for unknown point mutations in exonic sequences: cycling temperature capillary electrophoresis (CTCE)

**DOI:** 10.1186/1471-2156-8-54

**Published:** 2007-08-14

**Authors:** Per O Ekstrøm, Jens Bjørheim, William G Thilly

**Affiliations:** 1 Department of Surgical Oncology, The Norwegian Radium Hospital, Oslo, Norway; 2 The Medical Faculty, University of Oslo, Norway; 3 Department of Biological Engineering, Massachusetts Institute of Technology, Cambridge, Massachusetts, USA

## Abstract

**Background:**

Rapid means to discover and enumerate unknown mutations in the exons of human genes on a pangenomic scale are needed to discover the genes carrying inherited risk for common diseases or the genes in which somatic mutations are required for clonal diseases such as atherosclerosis and cancers. The method of constant denaturing capillary electrophoresis (CDCE) permitted sensitive detection and enumeration of unknown point mutations but labor-intensive optimization procedures for each exonic sequence made it impractical for application at a pangenomic scale.

**Results:**

A variant denaturing capillary electrophoresis protocol, cycling temperature capillary electrophoresis (CTCE), has eliminated the need for the laboratory optimization of separation conditions for each target sequence. Here are reported the separation of wild type mutant homoduplexes from wild type/mutant heteroduplexes for 27 randomly chosen target sequences without any laboratory optimization steps. Calculation of the equilibrium melting map of each target sequence attached to a high melting domain (clamp) was sufficient to design the analyte sequence and predict the expected degree of resolution.

**Conclusion:**

CTCE provides practical means for economical pangenomic detection and enumeration of point mutations in large-scale human case/control cohort studies. We estimate that the combined reagent, instrumentation and labor costs for scanning the ~250,000 exons and splice sites of the ~25,000 human protein-coding genes using automated CTCE instruments in 100 case cohorts of 10,000 individuals each are now less than U.S. $500 million, less than U.S. $500 per person.

## Background

Enumeration and discovery of statistically significant differences in the sum of all point mutations in the exons and splice sites of each known gene in large case and control cohorts can identify a large fraction of genes carrying inherited risk for common disease. This approach does not depend on the assumption of mono-allelic risk and is therefore independent of the method of linkage disequilibrium analysis [1]. Constant denaturing capillary electrophoresis (CDCE) permits separation and sensitive detection of all point mutations, known or unknown, within exon-sized target sequences and is to date the only mutation scanning technology demonstrated to permit unknown mutant enumeration in pooled DNA samples of 100 to 10,000 individuals [2,3]. In analyses employing CDCE, target DNA sequences are attached during high fidelity PCR to fluorescently labeled ~40–60 bp DNA sequences with melting temperatures of >94°C. These thermally stable sequences serve as "clamps" that prevent the dissociation during electrophoresis of the two anti-parallel stands of less thermally stable DNA (melting temperatures of 60–80°C) containing the target sequence. Simple melting and cooling of the PCR products converts rare mutant sequences into mutant/wild type heteroduplexes while capturing the numerically predominant wild type sequences as homoduplexes. The wild type homoduplexes are separated from all mutant-containing heteroduplexes based on differential average migration velocities on capillary gel electrophoresis at a column temperature optimized for each target sequence. Individual sequences are isolated as eluting peaks and subsequently identified by DNA sequencing.

However, CDCE separations are dependent on cooperative equilibria governing melting and annealing reactions in stringently defined target sequences comprising single isomelting domains, i.e. a DNA sequence of ~100 bp in which the melting temperature is essentially invariant with sequence (+/-0.15°C). CDCE does not detect all point mutations in target sequences that are not single isomelting domains; sequences comprising exons and adjacent intronic splice sites (exonic sequences) frequently comprise two isomelting domains or more irregular melting domains. The optimal CDCE separation temperature is generally found to be near the calculated melting temperature of the wild type homoduplex's isomelting domain. But satisfactory separation of *all *mutant sequences, especially the single base insertion or deletion mutations that minimally perturb the annealed heteroduplex, requires definition of optimal separation temperature within +/-0.1°C. Time-consuming trial and error optimization steps for each target sequence account for about 95% of the labor costs of assay for mutations in large human population samples [2,3]. Development of a set of CDCE assays for the multiple exons of a typical gene typically consume about four months for an experienced researcher. Furthermore, CDCE separations are excruciatingly sensitive to temperature variations requiring instruments capable of day-to-day reproducibility of +/-0.1°C. Under these conditions application of CDCE to the ~250,000 exons of the human genome would be a formidable task involving some 12,500 years of technical labor for optimization alone. Fortunately, these three technical limitations of CDCE have been overcome by the discovery that when the capillary temperature is cycled by several degrees Celsius, e.g. +/-6°C, that includes the calculated average melting temperature of any consensus target sequence, separations of homoduplexes and wild type/mutant heteroduplexes are achieved equivalent in peak resolution to optimized constant-temperature CDCE conditions [4-7]. The temperature cycling protocol obviates the need for stringent temperature control. Point mutations within target sequences with irregular melting domains are reliably detected. Most importantly, the desired degree of wild type-mutant separations is achieved without any prior optimization steps: the computer assisted design of the target sequence with attached high melting domain "clamp" is sufficient to define an analyte configuration suitable for separations on CTCE.

## Results

### Relationship of mean target melting temperature to optimal separation temperature

Each wild type and mutant sequence was amplified by PCR and approximately equal numbers of each molecule were mixed heated to melt the homoduplexes and cooled to form a mixture of the four expected duplexes: the wild type homoduplex, the mutant homoduplex and the two wild type/mutant heteroduplexes. Using these admixtures of DNA duplexes the effects on peak resolution of mean cycle temperature were studied relative to the average melting temperature of the wild type homoduplex.

**Figure 3:**
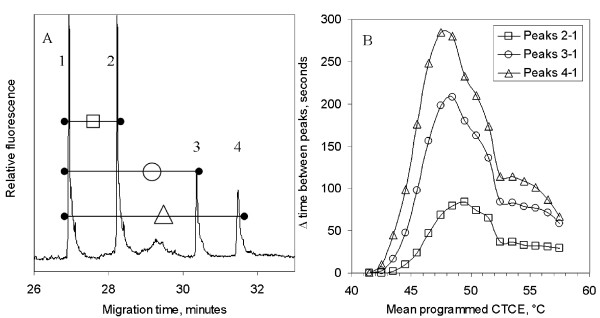
Illustration of peak resolution in CTCE. **A**. Separation of homoduplexes and heteroduplexes as electophoretic peaks using target sequence #1 from the gene BRCA1. The melting profile was irregular with a mean melting temperature of 70.7°C for the wild type homoduplex (peak 1). Twenty one-minute temperature cycles between 47°C and 59°C yielded baseline separations between homoduplexes and heteroduplexes. Small diffuse peaks result from errors generated during PCR with low-fidelity *Taq *polymerase and chemical reactions, such as thermal deamination of cytosine. These are avoided in mutation detection protocols that minimize the effect of deamination and employ Pfu DNA polymerase which does not copy passed a deaminated cytosine (uracil). **B. **Effect of mean column temperature on peak resolution. Temperature was varied in twenty one-minute cycles with 3°C amplitude and mean temperatures varying from 41.5 to 57.5°C.

Figure 3A shows as an example the degree of separation of the two polymorphic homoduplexes and the two heteroduplexes of target #1 derived from the gene *BRCA1*. The separation shown (Figure 3A) was achieved at the mean column temperature of 48.5°C in 7 M urea using twenty one-minute temperature cycles of amplitude 3°C. Target sequence #1 has a calculated mean melting temperature of 70.7°C at physiological salt conditions and about 49.7°C (70.7-21°C) in 7 M urea. Seventeen mean temperatures with 3°C amplitudes were tested in one degree Celsius increments from 41.5 to 57.5°C and the degree of separations observed as shown in Figure 3B. Separation was expressed as the number of seconds elapsed between any two peaks. The most rapidly eluting peak 1 contained the wild type homoduplex, peak 2 the mutant homoduplex and peaks 3 and 4 the two mutant/wild type heteroduplexes eluting in inverse order of mean melting temperature of the duplexes. At 41.5°C, no separations were observed. Significant separations of the wild type homoduplex and more stable heteroduplex (peak 3) were evident at 43.5°C, with the maximum separation observed at about 48.5°C. The degree of separation declined with an increase in mean cycle temperature from 48.5°C to 52.5°C, and then decreased more slowly up to 57.5°C, the maximum mean column temperature tested.

These data demonstrate that for target sequence #1 its mutant-containing heteroduplexes are separated by CTCE even when the mean temperature during cycling is deviates by +/-1.5°C from the optimal separation temperature. It appeared that the mean cycling temperature could be set close (+/-1.5°C) to the calculated mean melting temperature for a target wild type homoduplex to achieve near optimal separation of wild type homoduplex and derived mutant/wild type heteroduplexes. We subsequently tested this conclusion by extending our observations these observations to target sequences #2 through #12. All target sequences displayed separation optima near their calculated melting temperatures, with baseline separation of all mutant/wild-type heteroduplexes from the wild type homoduplexes within a range of +/-1.5°C of the optimum temperature for each target sequence. No laboratory optimization of separation conditions appeared to be required.

### Number and duration of cycles required for separation

**Figure 4:**
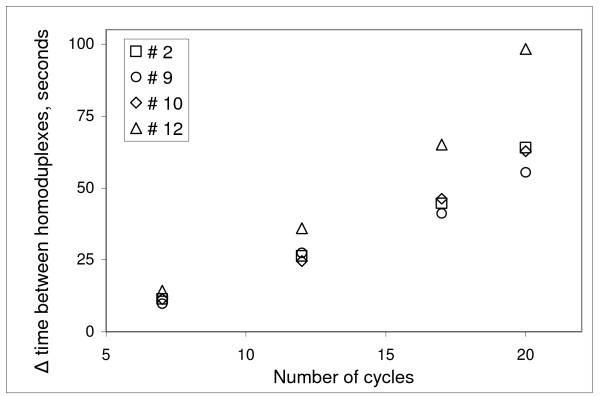
Separation of the wild type and mutant homoduplexes as a function of number (7, 12, 17 and 20) of one-minute cycles of 3°C amplitude (47°–50°C) for target sequences # 2, 9, 10 and 12.

We next explored reducing the number of temperature cycles with the aim of minimizing the time required per separation, a matter of importance in considering a pangenomic scanning effort. Accordingly, 7, 12, 17 and 20 one-minute cycles of 3°C amplitude were employed for fragments #2, 9, 10 and 12, using their mean calculated melting temperatures in urea (7 M) of 48.6, 48.2, 48.5, and 46.7°C, respectively. Using the time of separation between the less stable homoduplex and more stable mutant/wild type heteroduplex, we observed linear increases in the degree of separation of fragments #2, 9 and 12 with increasing cycle number, whereas fragment #12 displayed a somewhat supralinear relationship with cycle number as shown in Figure 4. As few as seven 1-min cycles of 3°C achieved a minimum 1-min separation of the less stable homoduplex and more stable heteroduplex. These observations that relatively few temperature cycles yielded a sufficient degree of separation may be of value in the performance of a truly high-throughput task, such as detecting all point mutations of the ~250,000 human exonic sequences in a million persons.

**Figure 5:**
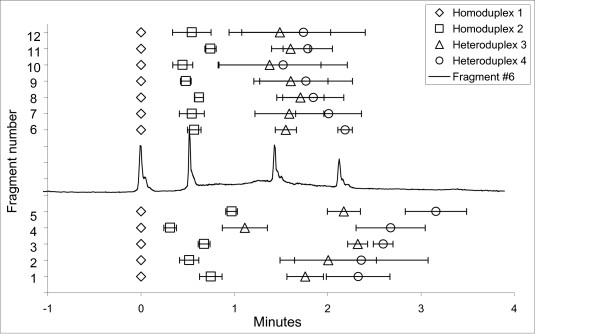
Differences in the CTCE migration times of all peaks relative to the most thermally stable homoduplex for target sequences #1–12. Five four-minute cycles (20 min) were employed with an amplitude of 12°C (47°C – 59°C). The results are illustrated as the average migration time difference +/- 1 standard deviation, n = 8. A representative electropherogram obtained from fragment #6 is incorporated to illustrate peak positions.

A high throughput parallel 10,000 capillary CDCE instrument, such as the one under construction at MIT, presents challenge of cycling temperature by 3°C in one minute by fluid flow in a relatively large (~5 l) capillary chamber. We therefore explored the tactic of increasing the amplitude of each cycle to 12°C, which reduced the number of cycles to five in a twenty-minute capillary separation. The data for octuplicate trials of each of twelve target sequences (#1–#12) separated in a capillary run five four-minute cycles of 12°C amplitude (47–59°C) are summarized in Figure 5. Under these easily created conditions we discovered that for fragments #1 through #12 that the separation of the less stable homoduplex from the more stable heteroduplex equaled or exceeded that obtained for twenty one-minute cycles.

### Separations of fifteen additional wild type/mutant exonic sequences without laboratory optimization

**Figure 6:**
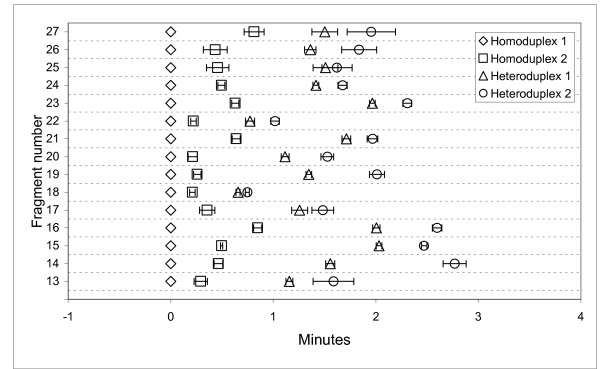
Differences in the CTCE migration times of all peaks relative to the most thermally stable homoduplex for target sequences #13–27. Five four-minute cycles (20 min) were employed with an amplitude of 12°C (48°C – 60°C). The results are illustrated as the average migration time difference +/- 1 standard deviation, n = 6.

To further test our preliminary conclusion that the degree of CTCE separation can be reproducibly achieved without resorting to any form of optimization except for calculating the target sequence melting temperatures, a second set of fragments (#13–27) was subjected to five four-minute cycles of 12°C amplitude (48–60°C). Data from sextuplicate runs in separate capillaries are presented in Figure 6 as in Figure 5. Capillary to capillary variation was marked, as is the case with capillary separations generally. This variation does not interfere with the automatic calculation of the ratio of the sum of peak areas for peaks eluting after the predominant wild type homoduplex peak to the sum of all peaks as an estimate of the fraction of mutant target copies in a pooled blood DNA sample. All four expected peaks were clearly resolved with baseline separations for every one of the twenty-seven target sequences in every run, including those of fragment #27 with a calculated melting temperature of 45.6°C, and fragments #17 and 19 with melting temperatures of 55.6°C in 7 M urea.

## Discussion

**Figure 1:**
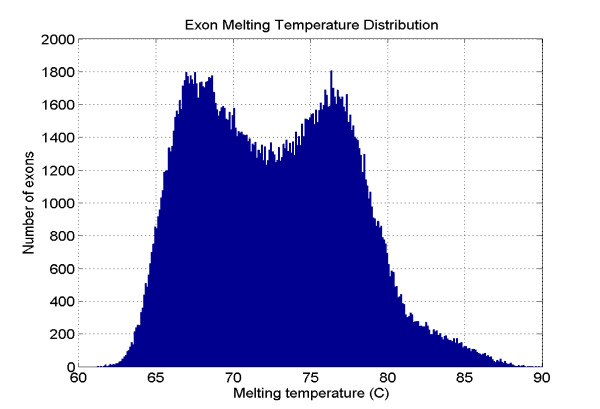
Distribution of mean melting temperatures of all known exonic sequences in the human genome. Based on the melting profiles created by a PubGene-MIT-Beckman Coulter collaboration, the histogram comprises the melting temperatures of all 236,039 exonic sequences from a set of human 27,561 protein encoding genes derived by Ensembl, a joint project of EMBL-EBI and the Sanger Institute. The resolution is 0.1°C. Mean temperatures were calculated for each exon plus 50 intronic base pairs 3' and 5' to each exon to include mRNA splice sites. (Reproduced with permission of PubGene, Inc.).

These observations demonstrate that cycling temperature denaturing capillary electrophoresis (CTCE) represents an important practical advancement in separation, detection and enumeration of unknown mutations in sequences of interest in the human genome. The degrees of separation obtained with the simple cycling temperature regimens are equivalent to those achieved using carefully defined optimal temperatures for each sequence in constant (temperature) denaturing capillary electrophoresis (CDCE). CTCE removes the practical impediment presented by the laboratory optimization requirements of CDCE and facilitates practical and economic pangenomic exon scanning to discover genes associated with inherited or somatic mutations conferring risk or common diseases. Secondly, CTCE is applicable to exonic sequences comprising two isomelting domains or an irregular melting profile (Figure 1; Figures 5, 6). So long as the target sequence is constructed such that the computationally defined melting profile consists of a monotonically decreasing melting temperature from the clamp junction through the ultimate primer sequence, CTCE yields the degree of separation required for scanning exonic sequences of 100–200 bp for unknown point mutations.

### Physical basis of CDCE and CTCE

The physical basis of denaturing gel electrophoretic separation of variant DNA sequences has yet to be established. Lerman and Fischer (1983) [8] initially applied the statistical mechanical reasoning of Poland [9] to the separation of mutants in a low melting domain naturally juxtaposed to a higher melting sequence serving as a "clamp" to achieve separate homoduplexes differing by a single base pair. In the initial Poland treatment duplexes could exist in only two states: melted and annealed. The separations observed with CTCE conditions appear to require a more complex explanation invoking multiple pathways for melting and re-annealing of a DNA duplex molecule and their interactions as the duplex is subjected to varying denaturing conditions while being pulled through a polymer matrix by a strong electric field [10]. Heteroduplexes, but not homoduplexes, contain multiple possible oligonucleotide runs, including a mismatch that destabilizes melting and annealing that is absent in homoduplexes. Heteroduplexes may thus maintain a significantly different time-average fraction in the slow-moving forms than homoduplexes throughout a large proportion of the temperature cycle under these non-equilibrium conditions.

### Practical application of CTCE in high-throughput tasks

We have recently reported the application of CDCE to analysis of exons, splice sites of all exons, and splice sites of two genes, CTLA4 and HBB, in large (~78,000 person) human population samples. The temperature optimization and stringent temperature control steps presented a major time and cost issue (~95% of labor costs) that would be severely limiting in a strategy that requires scanning the ~250,000 exonic sequences of the human genome [2,3].

Analysis of human somatic and/or inherited point mutations is important in both population genetics and clinical practice. In population genetics, such analyses are required to identify genes that carry mutations defining risks for a particular disease in families (rare diseases) or large general populations (common diseases). Once the genes and sets of risk-conferring mutations in a population are defined, clinical genetic analyses may be performed for individual patients to aid in diagnosis, determine the optimal form of therapy or provide more accurate prognosis.

Morgenthaler and Thilly [1] have argued that discovery of a gene or genes that carry point mutations conferring risk for common diseases may be achieved by pangenomic scans of the exons of all human genes in pair wise case-control cohort trials. Accounting for a series of confounding variables including multigenic and multiallelic conditions of risk they prescribed an effort to scan the exons and splice sites of ~25,000 human genes to enumerate mutations in population samples of 10,000 afflicted persons for each of the ~100 common diseases. This involves some 2.5 million pair wise gene-disease trials. To accomplish a pangenomic scan for inherited mutations associated with risk for common diseases in one million individuals, a method is required that effectively scans all the function-encoding domains of a gene, rarely fails to detect a point mutation, and permits high-throughput economical studies in a large number of genes and people.

Several methods have been devised to detect unknown DNA variants, based on differential migration velocities of mutant single-strand sequences or wild-type/mutant heteroduplexes drawn through a macromolecular matrix by an electric field [11-13]. Of these, only CDCE under optimized conditions has permitted the comprehensive detection of point mutations in pooled blood DNA samples from 100 to 10,000 persons [2,14].

Using an estimate of 400,000 target genomic sequences, CTCE scanning of case cohorts of 10,000 individuals each for the 100 most important human diseases with familial risk factors becomes a practical possibility. This involves scanning point mutations in 2 allelic copies per person × 10^6 ^persons × 400,000 exonic fragments per genome = 8 × 10^11 ^exonic fragments. Pooling DNA for each fragment from 100 persons within each case cohort would require about 4 × 10^9 ^capillary runs. With automatic loading and CTCE separation/gel replacement cycles of ~30 min, a well-engineered facility might be expected to achieve ~48 runs per capillary/day. With one hundred 10,000-capillary instruments ~4.8 × 10^7 ^pooled samples may be processed per day. The "task" of screening 4 × 10^9 ^pooled samples would require 4 × 10^9^/4.8 × 10^7 ^= 84 days. Ten instruments would complete the task in a more leisurely 840 days. Using either strategy, we estimate that collecting and processing samples from an ensemble of 100 disease-specific cohorts of 10,000 individuals each (including sample collection, sample processing, and CTCE analysis) can be performed for less than half a billion US dollars. This amounts to less than U.S. $500 per person to identify and enumerate the mutations carried by exons and splice sites of one million individuals distributed over any 100 important diseases that are possibly affected by inherited risk factors. The melting map for the entire human genome [15] is now available. CTCE makes it practical to scan for point mutations in the human population.

## Conclusion

CTCE provides practical means for economical pangenomic detection and enumeration of point mutations in large-scale human case/control cohort studies. We estimate that the combined reagent, instrumentation and labor costs for scanning the ~250,000 exons and splice sites of the ~25,000 human protein-coding genes using automated CTCE instruments in 100 case cohorts of 10,000 individuals each are now less than U.S. $500 million, less than U.S. $500 per person.

## Methods

### Choice of target sequences

We wished to choose target sequences to test the proposition that human exonic target sequences could be scanned for point mutations by CTCE without laboratory optimization steps. We began with the determination of the average melting temperatures of the known 236,069 exonic sequences of the human genome performed and reported by PubGene, Inc. (Oslo, Norway) shown as a distribution of the number of exons over average melting temperatures in Figure 1. Exonic sequences (exons + intronic splice sites) ranged from 60 to 88°C with some 93% having average melting temperatures less than 80°C, the upper limit of temperature at which DNA "clamps" are sufficiently stable to permit CDCE or CTCE separations [4]. Of the 7% of human exons with average melting temperatures greater than 80°C, more than half may be scanned by the expedient of dividing the target sequence so that all or most of the sequence may be scanned; we estimate that some 97% of the human exonic sequences may be interrogated by CTCE. (Note that the melting temperatures for near-physiological conditions are ~21°C higher than those observed in 7 M urea used in our experiments to allow separations at temperatures below 60°C.)

**Figure 2:**
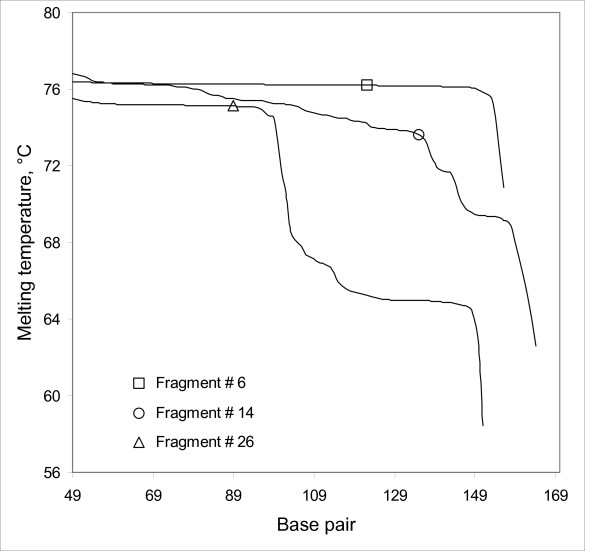
The melting profile of three target sequences # 6, 14 and 26 calculated with WinMelt illustrating a well-defined target of a single isomelting domain (#6), a target with two isomelting domains (#26) and a target with an irregular melting profile (#16). The symbols mark the position of the sequence differences AT>GC (#6), TA>CG (#14) and TA>CG (#26) in each target wild type>mutant pair in separation trials. The GC-clamp (~94°C) is not shown but was incorporated in the melting calculations attached to the higher melting temperature end of each target sequence.

We next had recourse to our library of human exonic sequences carrying a point mutation as a common polymorphism. We examined the set of several thousand possible exons and chose twenty seven simply on the basis of average melting temperatures ranging from ~66.6 to 78.1°C but without prior knowledge of the behavior on CTCE. Their identities, sequences and melting characteristics (WinMelt 2.0, Medprobe, Norway) are summarized in Table 1. By chance, of these twenty seven, six targets comprised a single isomelting domain (standard deviation in melting temperature among base pairs in the target sequence of less than 0.15°C) that would generally be expected to permit analysis by CDCE. The remaining twenty-one sequences comprised either more than one isomelting domain or an irregular melting profile with a standard deviation in target sequence melting temperature ranging from 0.3 – 4.2°C. Such sequences are in general not suitable for CDCE-based scanning. Figure 2 illustrates the variety of melting profiles represented within the set: fragment #6 comprised a single melting domain, fragment 26 comprised two isomelting domains differing by ~10°C while fragment #14 displays an irregular profile ranging in melting temperature over 7°C.

**Table 1:** Characteristics of 27 fragments used to test separation of CTCE

#	Gene symbol	NCBI rs number	DNA variant	PCR primer "Forward" 5'-3'	PCR primer "Reverse" 5'-3'	Mean melting, °C	Δ mean melting, °C	Fragment length, bp
1	BRCA1	rs799923	T59C	*gtccatggtgtcaagtttct	gttggacactgagactggtt	70.7	0.6	147
2	BRCA1	rs16940	T42C	*accccaaagatctcatgtta	cgagatactttcctgagtgc	68.9	0.4	154
3	MTHFR	rs1801133	T28C	*catccctattggcaggtt	aagaaaagctgcgtgatg	75.3	0.3	159
4	OPSIN	ac092402	T51G	*tctgtctttgctgcttcac	tttagaaaatgcctttggtc	70.6	0.1	159
5	MTHFR	rs1801131	A41C	*actccagcatcactcact	gagctgctgaagatgtgg	73.5	0.5	157
6	MTHFR	rs2274976	A35G	*ccaggttgaccaggaagt	gtgtaggacgaggccttt	75.7	0.3	156
7	CBS	rs234706	A45G	*ggtgactgaggtgtcagg	gacgcaccatcacactg	77.6	0.5	168
8	NQO1	rs1800566	T25C	*ctcatcccaaatattctcca	tctgtggcttccaagtctta	72.6	0.2	157
9	DPYD	rs3918290	A53G	*caccaacttatgccaattct	tgcatattggtgtcaaagtg	68.3	0.6	138
10	DPYD	rs17376848	T43C	*caccaacttatgccaattct	tgcatattggtgtcaaagtg	69.0	0.3	138
11	DPYD	rs1801265	T30C	*tcaggatttcttttccaatg	atcctcgaacacaaactcat	70.5	0.7	120
12	CTLA-4	rs5742909	T40C	*tcgaaaagacaacctcaag	aggaaattctccaagtctcc	67.8	0.3	175
13	COL1A1	rs1007086	A64G	*ctaaggatgggaggcacga	ccccctgtaagtatcactcc	76.5	0.2	132
14	COL1A1	rs1061237	T30C	*ttcctgtaaactccctccat	tgaaattgtctcccattttt	73.7	0.2	164
15	COL1A1	rs2857401	T54G	*ctgagatggcagttcttga	ctaaatgtctgttccctcca	74.1	0.2	155
16	COL1A1	rs2249492	T68C	*catagtgccctctctccat	gaggtcttggtggttttgta	76.0	0.4	161
17	COL1A1	rs2277632	A49G	*ctctccctccctcctactc	aatccagtactctcctgtgg	76.6	0.2	166
18	COL1A1	rs2075558	A48G	*catttttcatcaccgactg	agtaatggaggcaggaagat	75.8	0.2	168
19	COL2A1	rs2070739	A46G	*cagtgtacgtgaacctgcta	acctaccactgcaagaacag	76.5	0.3	168
20	COL2A1	rs2276454	T33C	*tccaggtcttcagggaat	tgagaggctgtaacctcagt	76.7	0.6	131
21	COL2A1	rs2276455	T61C	*ggtgagatgaaggaacagg	ctggtgatgaaggtttctgt	71.2	0.3	143
22	COL2A1	rs1635550	T57C	*agaagtacctttgcccaatc	caggaagaccctagacagaa	71.8	0.6	131
23	COL2A1	rs1635537	T94C	*agaaacttgctttgccttct	ctccttccctcctctgtact	72.5	0.3	166
24	COL2A1	rs1793958	T25C	*gatcttgagctcttcattgc	catgaggatatggaggtgac	71.9	0.3	142
25	COL11A1	rs2229783	T87C	*gtctgagtacccattggaaa	caagcagatgcagatgataa	67.3	0.3	157
26	COL11A1	rs3753841	T63C	*attctagggtcctgttggtt	aattggaaacattcactcca	70.2	0.2	151
27	COL11A1	rs2615987	T55G	*tgaatatgcacccttttctt	tgaacaccagaatttgaaca	66.6	0.4	155

For each target chosen, the consensus sequence and a known mutant sequence created by a single base-pair substitution were mixed, melted, and reannealed to create two homoduplexes and two heteroduplexes. For each target sequence, the designating number (#1, 2, 27), gene symbol, NCBI polymorphism reference number, specific mutation with its position in target fragment relative to the 5' end of the reverse primer, PCR primer sequences, average calculated melting temperature of the consensus homoduplex domain (including primer sequences), calculated change in melting temperature of the homoduplex due to the polymorphic base substitution and target fragment length without primers are shown. A thermally stable clamp sequence with a 5' fluorescent label (6-FAM) was synthesized separately for each test fragment incorporating the forward primer for each of the 27 target fragments. Clamp sequence: 6-FAM-CGCCC,GCCGC,GCCCC,GCGCC,CGTCC,CGCCG,CCCCC,GCCCG-forward primer.

In initial trials of CTCE, separations were unsatisfactory if a low melting domain of the target sequence were flanked by the clamp and a target sequence of higher melting temperature.

Thus, clamps were attached at the higher melting temperature end of sequences such as fragments #14 or #26 to avoid such a configuration. (A target sequence is occasionally encountered with a low melting domain flanked by higher melting temperature sequences. Such target sequences are scanned by the expedient of scanning them as two separate sequences both terminating in the low melting domain.)

### DNA samples and PCR

Genomic DNA was extracted from anonymous blood donor samples with a QIAamp DNA Blood Midi Kit (Qiagen Inc., Valencia, CA, USA). Primers were designed with Primer3 software [16] so that all fragments could be amplified under similar PCR conditions. A 40 base-pair sequence with a melting temperature of ~94°C and labeled with 6-FAM was incorporated into one primer during oligonucleotide synthesis, creating a high melting domain "clamp" adjacent to the target sequence (Table 1). Amplification was performed on a PTC-200 thermal cycler (MJ research, Waltham, MA, USA) by mixing ~50 ng genomic DNA with 2.5 mmol of each dNTP (Perkin Elmer, Oslo, Norway), 10 × *Taq *buffer, 1 unit *Taq*, 0.1 units of *Pfu *and 5 pmol of each primer (MedProbe, Oslo, Norway) in a final volume of 20 μl. The cycling parameters included 35 cycles of denaturation for 30 sec at 94°C, annealing at 56°C for 30 sec and elongation at 72°C for 1 min, followed by an elongation step of 72°C for 10 min at the end of the last cycle.

### Electrophoresis

A 96-capillary DNA analyzer, MegaBACE™ 1000 DNA Analysis System (Amersham Biosciences, Uppsala, Sweden), was adapted for CTCE separations with software modifications to control temperature cycling. The distance from the anode to detector was 40 cm. Linear polyacrylamide (MegaBACE LPA) containing 7 M urea was replaced in capillaries prior to each run. Samples were loaded into the capillaries from 96-well plates by electrokinetic injection at 133 V/cm for 12 seconds. Electrophoresis was performed at a constant field of 133 V/cm. Laser-induced fluorescence was used with excitation at 488 nm (blue argon laser) and emission at 520 nm (FAM channel).

### Modification of MegaBACE to allow for high temperature settings

The instrument was modified by replacing the "tmpr.nxe" file with an updated version obtained from Molecular Dynamics (acquired by Amersham Biosciences, which is now part of GE Healthcare). The file allows for disconnecting the cooling fan, and facilitates changes to the temperature limits in the registry. Temperature changes, which made cycling possible, were made in the "macro.ini" file under the section [Inject Samples and Run]. Files and detailed descriptions are available upon request.

### Denaturing conditions and temperature control

The denaturing temperature in the capillary chamber, i.e. cycling temperature, was programmed in the macro.ini file of the Instrument Control Manager (ICM) software package (Amersham Bioscience). Files are available from the corresponding author upon request. The MegaBACE instrument permitted a heating/cooling rate of about 0.1°C/second. At this rate of temperature change, we could observe the degree of separation as a function of cycle number (5 to 20), mean temperature (41.5 to 57.5°C) and temperature cycle amplitude (3°C to 12°C). Due to the fixed temperature ramping rate (~0.1°C/sec), the effective on-column separation time varied as a function of cycle number and amplitude. Hence, a temperature range of 3°C created a 60 second cycle, whereas a cycle with a temperature range of 12°C created a 240 second cycle. The MegaBACE Sequence Analyzer software program (Amersham Bioscience) was employed to measure the migration times and areas of all peaks.

## Authors' contributions

POE carried out the allele separation on the MegaBACE and wrote the cycling conditions in the macro.ini file. POE and JB evaluated all the electropherograms and performed the calculations. WGT participated in the design of the study and performed the statistical analysis. All authors contributed equally in the writing of the manuscript and have read and approved the final version.
